# Exploration of Early-Treatment-Associated Changes in Metabolic and Inflammatory Biomarkers in First-Episode Psychosis in Italian Patients

**DOI:** 10.3390/ijms27042065

**Published:** 2026-02-23

**Authors:** Elisabetta Maffioletti, Clarissa Ferrari, Roberta Zanardini, Roberta Rossi, Sarah Tosato, Chiara Bonetto, Mario Ballarin, Antonio Lasalvia, Mirella Ruggeri, Massimo Gennarelli, Andrea Geviti, on behalf of the GET-UP Group, Luisella Bocchio-Chiavetto

**Affiliations:** 1 Department of Theoretical and Applied Sciences (DiSTA), eCampus University, 22060 Novedrate, Italy; elisabetta.maffioletti@uniecampus.it (E.M.); luisella.bocchiochiavetto@uniecampus.it (L.B.-C.); 2 Psychiatry Unit, IRCCS Istituto Centro San Giovanni di Dio Fatebenefratelli, 25125 Brescia, Italy; rrossi@fatebenefratelli.eu; 3 Fondazione Poliambulanza Istituto Ospedaliero, 25124 Brescia, Italy; clarissa.ferrari@poliambulanza.it; 4 Clinical Laboratory, ASST-Valcamonica, 25040 Esine, Italy; roberta.zanardini@asst-valcamonica.it; 5 Section of Psychiatry, Department of Neuroscience, Biomedicine and Movement Sciences, University of Verona, 37129 Verona, Italy; chiara.bonetto@univr.it (C.B.); mario.ballarin@univr.it (M.B.); mirella.ruggeri@univr.it (M.R.); 6 Center for Medical Sciences (CISMed), University of Trento, 38122 Trento, Italy; antonio.lasalvia@unitn.it; 7 Department of Molecular and Translational Medicine, University of Brescia, 25121 Brescia, Italy; massimo.gennarelli@unibs.it; 8 Genetics Unit, IRCCS Istituto Centro San Giovanni di Dio Fatebenefratelli, 25125 Brescia, Italy; 9 Service of Statistics, IRCCS Istituto Centro San Giovanni di Dio Fatebenefratelli, 25125 Brescia, Italy; ageviti@fatebenefratelli.eu

**Keywords:** first-episode psychosis (FEP), psychosocial intervention, cognitive–behavioural therapy, CBT, inflammation, metabolism, leptin, ghrelin, glucagon-like peptide-1, GLP-1

## Abstract

Studies conducted in first-episode psychosis (FEP) patients have shown alterations in inflammation and metabolism. Our objective was to investigate potential treatment-related effects on these systems in Italian FEP patients undergoing either an experimental treatment consisting of a multi-element psychosocial intervention (EXP), including cognitive–behavioural therapy, or treatment as usual (TAU). A total of 191 FEP patients with first contact between April 2010 and March 2011 were clinically assessed at baseline and after 9 months of treatment, and the serum levels of 19 analytes were determined through single or multiplex enzyme-linked immunosorbent assays (ELISAs). A significant increase was observed in leptin levels and a significant decrease in Glucagon-Like Peptide-1 (GLP-1) levels during the treatment (time effect, *p* < 0.001 for both), with no significant interaction between time and treatment type. Although ghrelin levels changed significantly over time in the whole cohort (*p* = 0.008), a significant decrease was observed only in the EXP group (post hoc test: *p* = 0.001). None of the biomarkers measured at baseline showed a predictive effect on treatment efficacy, and no significant associations were identified between changes in clinical scores and changes in biomarker levels. These results suggest that early-phase psychosis treatments are associated with possible effects on metabolic regulation.

## 1. Introduction

In major psychoses, studies on molecular markers, together with innovative clinical tools and other kinds of biomarkers, could help the development of precision medicine strategies to improve diagnostic accuracy and treatment response, through individually tailored therapies [1,2,3,4]. The potential usefulness of research in this field is especially high at the onset, and in particular in first-episode psychosis (FEP) patients, who are minimally treated or untreated, to define the optimal therapeutic approach [4,5]; indeed, delayed or inadequate treatments may lead to worse functional outcomes, permanent alterations, and psychosis chronicity [6,7].

Early research on the prediction of treatment response conducted in FEP patients over the past decades reported some promising results identifying pharmacogenetic markers and other molecular markers linked to immune-inflammatory and metabolic functions [8,9], and consistent evidence has indicated alterations in inflammatory response and metabolic homeostasis in psychotic disorders, from the initial phases of the disease.

Studies in FEP patients, also involving drug-naïve subjects, described altered levels of several cytokines, chemokines, and immune modulators [10,11,12,13,14], suggesting the presence of an inflammatory syndrome at the psychosis onset, associated with the symptomatology. Through a machine learning approach, in 2021, our research group identified a transcriptional signature involving immunity genes able to distinguish FEP patients from both chronic psychotic patients and unaffected controls [15] and demonstrated the existence of an “inflammatory” subgroup of FEP patients characterised by a multivariate pattern of immunomarkers involved in inflammatory activation [16]. The inflammatory imbalance in FEP patients could also activate compensatory mechanisms, including changes in the expression and signalling of neurotrophins, involving the Brain-Derived Neurotrophic Factor (BDNF) and the Vascular Endothelial Growth Factor (VEGF) [17,18,19,20,21].

Together with alterations in inflammatory response, psychotic patients show an increased risk of developing cardiovascular diseases and type 2 diabetes compared to the general population [22,23,24]. Studies in FEP patients highlighted abnormal glucose and lipid metabolism and appetite regulation [25,26,27,28]. In particular, evidence has reported a decrease in insulin sensitivity and an increase in serum insulin levels, insulin resistance indices (HOMA1-IR and HOMA2-IR), blood glucose and triglycerides, as well as abnormal levels of appetite-regulating hormones, also in adolescents and antipsychotic drug-naïve patients [29,30,31,32,33,34,35]; these alterations were also associated with symptomatology features by some studies. Moreover, insulin resistance was indicated as an early marker of increased vulnerability to weight gain and abdominal obesity during the first year of treatment, as reported in a study published in 2015 [36].

During the initial phases of treatment, modulations in inflammatory and metabolic markers were reported in FEP patients [36,37,38,39]. Moreover, the baseline levels of various immune and metabolic markers were also described as predictive of treatment response, and their changes were correlated with clinical outcomes [39,40,41,42,43,44,45,46,47].

In a previous study by our research group [48], published in 2018 and conducted in the framework of the project “Genetics, Endophenotypes and Treatment: Understanding Early Psychosis” (GET UP—study protocol published in 2012) [49], we reported alterations of 10 markers associated with immune regulation, growth factors and glucose metabolism in FEP patients before treatment. In particular, MIP-1b/CCL4, VEGF, IL-6 and PAI-1 serum levels were significantly higher, while IL-17, ghrelin, glucagon and GLP-1 were lower in FEP patients compared to unaffected control subjects. No differences were evidenced among the different diagnostic subgroups for these markers. The receiver operating characteristic (ROC) analysis showed that MIP-1b/CCL4, ghrelin and glucagon had the best discriminant power between cases and controls; moreover, alterations of these three markers were also present in the subgroup of drug-naïve patients.

More in depth, the GET UP project was constituted by four partner projects, including the clinical trial “Psychosis Early Intervention and Assessment of Needs and Outcome” (GET UP PIANO) and the project “Genetic data Utilization and Implementation of Targeted Drug Administration in the Clinical Routine” (GET UP GUITAR). The GET UP PIANO project consisted in a cluster-randomised controlled trial, comparing an add-on experimental integrated multi-element psychosocial intervention, comprising cognitive–behavioural therapy (CBT), family intervention, and case management (EXP arm), with treatment as usual (TAU arm) for FEP patients in 117 Community Mental Health Centres (CMHCs) in a large area of Northern/Central Italy (10 million inhabitants). FEP patients were followed during their treatment for 9 months, evaluating the efficacy of the multicomponent intervention on symptom improvement and reduction in in-hospital stay. The results, published in 2015, indicated that patients in the experimental arm had a greater reduction in overall symptom severity, while no difference was found for the duration of hospitalisation [50].

On the basis of the above-presented evidence, the aim of this exploratory study, located in the framework of the GET UP GUITAR project, was to explore, in FEP patients, treatment-related effects across multiple aims, by analysing a relatively large panel of 19 candidate serum markers involved in inflammation and metabolism. This approach also aimed to provide insights into the possible interplay between metabolic and inflammatory factors during early treatment in FEP patients.

The specific objectives were: (1) the identification of possible baseline molecular markers predictive of clinical outcome after the first 9 months of treatment, represented by an experimental multi-element psychosocial intervention including CBT, in addition to routine care (EXP group) or by treatment as usual (TAU group), and (2) the detection of possible molecular changes after 9 months of treatment, also considering their association with clinical response. Although the biological samples were collected in 2010–2011, the study remains relevant because there is still a limited number of studies addressing this specific topic.

## 2. Results

### 2.1. Baseline Socio-Demographic Characteristics, Clinical Features and Biomarker Levels

Descriptive statistics for the socio-demographic characteristics and the body mass index (BMI) of the whole study cohort (*n* = 191), as well as of the EXP (*n* = 128) and TAU (*n* = 63) groups, are shown in Table 1, along with the results of the corresponding statistical tests. Since the two treatment groups differed for sex and age of onset, subsequent analyses involving comparisons between these groups were corrected for these variables.

The baseline concentrations (T0) of the 19 biomarkers analysed are depicted in Table 2.

The scores of the clinical scales before treatment (T0), including the Positive and Negative Syndrome Scale—PANSS [51], the Hamilton Rating Scale for Depression—HAM-D [52] and the Global Assessment of Functioning—GAF [53], are reported together with those after treatment (T1) in the following paragraphs (Table 3). The duration of untreated psychosis (DUP), which is a known predictor of treatment success [7], did not significantly differ between EXP and TAU patients (EXP: 6.6 ± 14.1 months, TAU: 6.3 ± 17.8 months, *p* = 0.917).

### 2.2. Associations of Baseline Socio-Demographic and Clinical Features with Baseline Biomarker Levels

We identified significant, although low-to-moderate, correlations between baseline biomarker levels and socio-demographic, anthropometric and clinical features. In particular, the body mass index (BMI) was positively correlated with concentrations of insulin (*p* = 0.050, Spearman’s coefficient ρ = 0.18), leptin (*p* = 0.033, ρ = 0.20) and resistin (*p* = 0.026, ρ = 0.20). Moreover, age of onset was positively correlated with levels of BDNF (*p* = 0.003, ρ = 0.21), PAI-1 (*p* = 0.017, ρ = 0.18) and leptin (*p* = 0.026, ρ = 0.17). Significant differences were also detected according to sex for leptin (*p* < 0.001) and visfatin (*p* = 0.002). Concerning the clinical characteristics, significant negative correlations were observed between PANSS mean total scores and both BDNF (*p* = 0.002, ρ = −0.22) and leptin (*p* = 0.021, ρ = −0.17), between HAM-D scores and BDNF (*p* = 0.040, ρ = −0.15), and between DUP and ghrelin (*p* = 0.042, ρ = −0.16).

### 2.3. Clinical Changes Across the 9-Month Treatment

After 9 months of treatment (T1), the mean total PANSS, HAM-D and GAF scale scores showed significant changes (time effect: *p* < 0.001 for all three): on average, patients’ symptomatology had improved.

Significant interaction (time × treatment) effects were also detected (mean total PANSS: *p* = 0.008; HAM-D: *p* = 0.010; GAF: *p* = 0.035), with a more pronounced improvement in the EXP group compared to the TAU group.

At the end of the 9 months of treatment, the percentage of patients taking antipsychotic drugs did not significantly differ between the two treatment groups (EXP: 64.8%; TAU: 65.1%; *p* = 0.363).

### 2.4. Biomarker Changes Across the 9-Month Treatment

Among the 19 biomarkers that were analysed, significant results were detected for leptin, ghrelin and Glucagon-Like Peptide (GLP-1) according to GLMM analyses.

Due to the skewed distribution of these variables, they were modelled using a Gamma distribution with a log link, and no direct log transformation of the data was applied. All the models converged successfully, and residual diagnostics revealed no significant violations (all tests, including residuals versus fitted values, KS, dispersion, and outlier tests, *p* > 0.05). Estimated marginal means were computed on the link scale and subsequently back-transformed to the original scale. Effect sizes for changes over time were derived from exponentiated contrasts and expressed as percentage changes, calculated as (exp(β) − 1) × 100. Corresponding 95% confidence intervals were obtained by back-transforming the confidence limits of the model-based contrasts.

**Figure 1:**
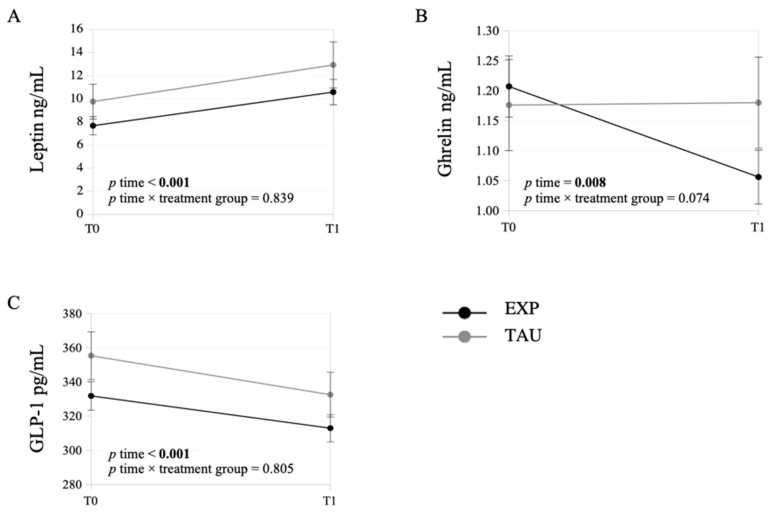
Modifications from baseline (T0) to the end of the 9-month treatment (T1) in the serum levels (estimated marginal means ± standard errors) of leptin (**A**), ghrelin (**B**) and GLP-1 (**C**) in the EXP (black line) and TAU (grey line) groups.

A significant increase was found for leptin after treatment (time effect: *p* < 0.001). This modulation did not differ according to the treatment group (EXP vs. TAU, time × treatment effect: *p* = 0.839), and the extent of increase was similar in the two groups (EXP: +37.9%, 95% CI = [+11.4%, +71%], *p* = 0.003; TAU: +32.5%, 95% CI = [−3.7%, +81%], *p* = 0.084) (Figure 1A).

Ghrelin was also observed to significantly change over time (time effect: *p* = 0.008), with an almost-significant time × treatment effect (*p* = 0.074). In particular, ghrelin decreased by 12.4% (95% CI = [−5%, −19.4%]) in the EXP group (*p* = 0.001) and did not change in the TAU group (*p* = 0.966) (Figure 1B).

Finally, GLP-1 decreased across time (time effect: *p* < 0.001) irrespective of treatment group (time × treatment effect: *p* = 0.805). GLP-1 decreased by 5.7% (95% CI = [−2.5%, −8.8%]) in the EXP group and by 6.4% (95% CI = [−1.5%, −11.1%]) in the TAU group (EXP: *p* < 0.001, TAU: *p* = 0.012) (Figure 1C).

Comprehensive results are reported in Table 4.

Moreover, we repeated the analyses, including a random intercept for CMHC to assess the extent of variability explained by the recruiting centre. Likelihood ratio tests comparing models with and without the CMHC random intercept showed no significant results (*p* = 0.95 for leptin, *p* = 1 for ghrelin, and *p* = 0.223 for GLP-1), indicating that CMHC does not contribute meaningful variability in these analyses.

Finally, for all the considered biomarkers, no significant interaction was observed between time and antipsychotic, mood stabiliser, antidepressant, or anxiolytic treatment status (i.e., whether patients were taking these drugs or not), as assessed by the GLMMs.

### 2.5. Relationships Between Biomarkers and Clinical Changes Across Treatment

No biomarker measured at baseline showed a significant predictive effect for any of the considered clinical scales.

Also, regarding the possible correlations between changes in clinical scale scores and those in the levels of biomarkers across treatment, no significant result was found.

## 3. Discussion

Our study aimed to explore the molecular effects of treatment in FEP patients by analysing a panel of 19 candidate serum markers involved in inflammation and metabolism.

Whereas no baseline molecular marker was identified as a predictor of clinical outcome after 9 months of treatment, different metabolic markers showed significant changes, including leptin, GLP-1 and ghrelin.

Leptin increased after 9 months in both treatment groups (EXP and TAU), without any significant difference attributable to the treatment group. Similarly, Glucagon-Like Peptide (GLP-1) showed a decrease across time in both treatment groups, with no treatment group-related difference. For ghrelin, in contrast, almost significant between-group differences emerged. The overall time effect identified by the GLMM was largely attributable to the EXP group, where ghrelin levels showed a significant decrease over time, while they remained stable in the TAU group.

The modulation of leptin, ghrelin and GLP-1 after 9 months of treatment underscores that the evaluated therapies for psychosis conducted in the early phase after onset may influence the metabolic system at different levels.

Leptin is a hormone produced by the adipose tissue, and its peripheral levels typically mirror the quantity of adipose tissue in the body. Leptin acts to increase metabolic rate and to reduce appetite; it plays an important role in various peripheral tissues and can enter the brain via saturable receptor-mediated transport across the blood–brain barrier or via cerebrospinal fluid. Leptin can also be synthesised and released locally, since leptin mRNA has been detected in the central nervous system [54,55,56,57]. Leptin binds to its receptors located in several regions of the hypothalamus, where it acts to suppress food intake. Research has revealed that leptin receptors are not limited to the hypothalamus, but they are also present in various other brain areas, including the amygdala, hippocampus, cerebellum, medulla, neocortex and basal ganglia [58,59]. This widespread distribution has prompted investigation into leptin’s broader roles beyond metabolism. For instance, its presence in the hippocampus has encouraged studies into its potential involvement in learning and memory, while receptors found in the amygdala have suggested possible links to emotional disorders [60,61,62,63,64,65].

Under physiological conditions, leptin can interact with its hippocampal and cortical receptors, activating pro-survival signalling pathways such as JAK2-STAT3, PI3K-Akt and MAPK. This activation enhances synaptic plasticity and long-term potentiation, while simultaneously mitigating oxidative stress. Leptin is also able to modulate neuroinflammation by promoting pro-inflammatory cytokine secretion and interacting with dopamine neurotransmission [66,67]. Several studies have demonstrated possible roles of leptin in various psychiatric illnesses going from eating disorders to affective and psychotic disorders [68,69,70].

Studies comparing leptin levels in FEP patients to controls have produced contrasting results [34,48,71,72], and a meta-analysis conducted in 2019 has found no overall difference except for decreased levels in drug-naïve patients [32]. Low leptin levels in FEP have also been associated with specific aspects of the illness phenotype, such as language deficits [71] and suicidal behaviour [73], based on reports published in 2020 and 2019, respectively.

Several studies have attempted to determine the effects of antipsychotics on leptin. A meta-analysis published in 2015 showed that leptin increases during treatment with olanzapine, clozapine and quetiapine, whereas for haloperidol and risperidone, no significant modifications were detected. In the same study, changes in the body mass index have been associated with an increase in leptin levels, suggesting that this increase is most likely due to weight gain during treatment rather than to a direct effect of antipsychotics on its production [74]. On the other hand, both weight gain and higher leptin plasma levels have been associated with clozapine response in schizophrenic patients [75], suggesting that this “side effect” mediated by leptin modulation may also have a role in the therapeutic effect. In this regard, a systematic review published in 2018 also reported an overall positive association between antipsychotic-induced weight gain and clinical response in more than 70% of the reviewed studies [76].

As previously outlined, together with its well-known metabolic effects, leptin also participates in brain remodelling and in cognitive and emotional regulation; thus, the observed increase in its levels in our study sample could be part of responses to antipsychotic drugs. Among the possible mechanisms, major roles could be played by leptin interaction with BDNF and dopamine neurotransmission. Indeed, treatment with leptin has been shown to increase BDNF mRNA levels in the hippocampus of mice, through an epigenetic mechanism that involves histone modifications mediated by the stimulation of AKT signalling. Moreover, as reported in a study published in 2003, leptin receptors are widely expressed on dopamine neurons in brain regions like the ventral tegmental area and the substantia nigra [77], and leptin is able to regulate the dopamine transporter expression, suggesting that this hormone can modulate dopaminergic neurotransmission in the brain [67].

GLP-1 is an incretin hormone that plays a key role in regulating blood glucose levels and lipid metabolism, produced by the cleavage of proglucagon and released by intestinal L-cells in response to meals. GLP-1 enhances insulin secretion and inhibits glucagon release, delays gastric emptying and reduces food intake by suppressing central appetite centres [78,79]. GLP-1 receptor agonists, also known as “incretin mimetics”, are a class of medications licensed for the treatment of type 2 diabetes mellitus and obesity. These drugs mimic the action of endogenous GLP-1, making them powerful tools to control blood glucose and to improve metabolic syndrome. Moreover, neuroprotective and anti-inflammatory properties, together with the regulation of reward pathways, have recently emerged as additional modes of action for GLP-1 agonists, and emerging consensus indicates that these drugs could be repurposed for use in several neuropsychiatric conditions, including psychoses [80]. Systematic reviews and meta-analyses indicate that these therapeutic agents are effective in mitigating antipsychotic-associated body weight gain and improving metabolic parameters in patients treated with antipsychotics [81,82,83]; in this regard, clozapine has also been observed to directly reduce GLP-1 levels in obese rats, contributing to impaired glucose tolerance, as described in a study published in 2009 [84]. Returning to GLP-1 peripheral levels in relation to psychoses, a previous study by our research group published in 2018 highlighted decreased serum levels of GLP-1 in the same FEP patients included in the present study (only a small proportion of whom were drug-free) compared to controls [48]. Concerning specific treatment-induced metabolic effects, which have not been explored in the present study, higher GLP-1 levels have been previously associated with more marked weight gain and other indices of metabolic syndrome in male, but not in female, patients with schizophrenia undergoing clozapine treatment, as shown in a study published in 2021 [85]. In light of this, the decrease in GLP-1 during the first 9 months of treatment in FEP patients observed in the present study may reflect two non-mutually exclusive mechanisms. First, GLP-1 reduction could be related to the disease itself and its progression, independent of treatment. Second, the decrease in GLP-1 could represent an effect of antipsychotic treatment, consistent with preclinical evidence indicating that antipsychotics can suppress GLP-1 production. Overall, these findings highlight the role of GLP-1 dysregulation as an early contributor to metabolic vulnerability in FEP and support further exploration of interventions targeting this pathway.

Ghrelin is an acylated peptide hormone known as one of the main appetite-regulating hormones; it is synthesised by endocrine cells of the gastric mucosa and activates hypothalamic orexigenic neurons to promote meal initiation and food-seeking behaviour. Receptors for ghrelin are distributed in various regions of the central nervous system, such as the amygdala, hippocampus, nucleus accumbens and ventral tegmental area, where this hormone can affect multiple neurotransmitter systems, including the dopamine, norepinephrine, and serotonin ones [86]. Thus, it is not surprising that ghrelin is increasingly implicated in complex cerebral functions and in related psychiatric conditions such as stress response and anxiety or depression [87]. It has also been highlighted that individuals with schizophrenia exhibit altered peripheral levels of ghrelin compared to non-affected subjects, and that antipsychotic medications can influence ghrelin concentrations, although the direction of these effects remains contradictory across studies, possibly due to their heterogeneity [71,86]. These observations may, at least in part, reflect the ability of ghrelin to enhance dopamine secretion and to modulate dopamine receptor gene activity [86], potentially contributing to the dopaminergic dysregulation underlying this disorder and to the effects of antipsychotic treatments. In FEP patients, according to a meta-analysis based on three studies, ghrelin levels have been reported to be unaltered in antipsychotic-naïve or minimally medicated FEP patients [32]. One of the studies included in this meta-analysis was performed by our research group in 2018 and reported decreased concentrations of ghrelin in FEP patients compared to controls [48]. Concerning the results of the present study, given that the EXP and TAU groups did not differ in whether patients were receiving antipsychotic treatment or not, it can be hypothesised that the reduction in ghrelin levels only among patients who received the experimental treatment may reflect an attenuation of stress, which has been described to be associated with elevated ghrelin levels as reported in a systematic review published in 2021 [88]. By improving coping strategies, reducing perceived stress, and enhancing social functioning, psychosocial interventions may normalise the activity of the hypothalamic–pituitary–adrenal axis and restore adaptive neuroendocrine balance. Thus, decreased ghrelin in patients who underwent the experimental treatment could represent a biomarker of improved stress regulation rather than a direct indicator of symptom remission, an effect not observed in patients receiving treatment as usual, who, despite showing clinical improvement, likely do not exhibit this effect on stress regulation. However, it cannot be excluded that the observed differential effect between the two treatment groups may also have been influenced by differences in the types, number and dosage of antipsychotics and/or other psychotropic drugs that patients were taking. Since psychotropic treatments were assessed using broad yes/no categories, this study does not fully capture possible variability related to specific compounds, dosing, treatment changes, or metabolic risk profiles, which may represent a source of confounding. Moreover, patients were followed over a relatively long period, during which pharmacological treatment often changed, and this would have made it particularly challenging to accurately model the effects of all combinations and changes in medication. Therefore, the precision with which treatment-related effects on metabolic regulation can be interpreted is limited, and future studies specifically addressing these aspects are warranted.

Other important limitations should be considered. First, in the absence of concurrent comprehensive metabolic endpoints, it is not possible to definitively attribute the observed biomarker changes to treatment rather than to weight gain or metabolic drift related to the underlying pathology; these results should, therefore, be interpreted with caution. Second, only baseline and 9-month follow-up measurements have been performed. While these two time points can provide some insight into possible treatment-associated changes, they are likely insufficient to capture the full complexity of biological dynamics. By relying only on baseline and 9-month data, the study cannot adequately characterise the trajectories of these rapidly evolving biomarkers, and it cannot identify, for example, possible transient changes. Future studies with more frequent sampling in the initial treatment period would be essential to better understand the temporal evolution of these biological responses.

Finally, the date of first contact for participants in the study was between 2010 and 2011. Although the data are not recent, the findings remain relevant to current research questions.

## 4. Materials and Methods

In the context of the GET UP PIANO clinical trial, FEP patients were recruited and assessed at baseline and after 9 months of treatment with a set of standardised instruments. Details on the study protocol and on the clinical results are provided elsewhere [37,38]. The GET UP GUITAR project, focused on the identification of molecular markers and genetic variants associated with psychosis onset and response to medication, was conducted on the enrolled sample of the GET UP PIANO clinical trial, and blood samples were collected from patients who consented to participate in these biological studies. This manuscript is based on data derived from the biochemical analyses of the GET UP GUITAR project, obtained using the serum of FEP patients at admission to the GET UP PIANO clinical trial (baseline, T0) and after 9 months of treatment (follow-up, T1). The GET UP research program was approved by the Ethics Committee of the coordinating centre (Azienda Ospedaliera Universitaria Integrata di Verona—protocol n. 1682, 20 May 2009) and of each participating unit.

### 4.1. Study Participants

The initial sample group included individuals with potential psychosis who had a first contact with a CMHC during the index period (1 April 2010–31 March 2011, with last contact by 2012). Inclusion criteria for FEP were as follows: (a) aged 18–54 years; (b) residence within the catchment areas of CMHCs; (c) presence of at least one of the following symptoms—hallucinations, delusions, qualitative speech disorder, qualitative psychomotor disorder, and bizarre or grossly inappropriate behaviour—or at least 2 of the following symptoms—loss of interest, initiative, and drive, social withdrawal, episodic severe excitement, purposeless destructiveness, overwhelming fear, and marked self-neglect; and (d) first lifetime contact with CMHCs, prompted by these symptoms. Exclusion criteria were the following: (a) pre-existing anti-psychotic medication (duration > 3 months) prescribed by psychiatric or other medical agencies for a mental disorder identical or similar to the current one; (b) mental disorders due to a general medical condition; (c) moderate-to-severe intellectual disability assessed by clinical functional assessment; and (d) psychiatric diagnosis other than ICD-10 for psychosis. Written informed consent was obtained from all the participants prior to any study procedures, during the period of April 2010 to March 2011. Participants were provided with a detailed explanation of the study objectives and procedures and were given the opportunity to ask questions before signing the consent form.

In total, within the GET UP PIANO clinical trial, 444 FEP patients were recruited and evaluated at baseline, as explained in detail in the following paragraph, “Clinical Assessment”. Among these, 191 gave consent for blood sampling for the GET UP GUITAR project and completed the study with the follow-up clinical assessment at T1. Ninety-four patients underwent blood sampling also at T1. A flow diagram for the biomarker study, including per-analyte sample sizes, is provided in Supplementary File S2.

After clinical stabilisation, FEP patients were assessed with a comprehensive set of clinical measures. A set of core outcome instruments, including the Positive and Negative Syndrome Scale—PANSS, which evaluates the severity of positive, negative and general symptoms associated with psychotic disorders [51], the Hamilton Rating Scale for Depression—HAM-D, which assesses the severity of depressive symptoms [52], and the Global Assessment of Functioning—GAF, which measures the overall psychological, social, and occupational functioning [53], was administered by a panel of 17 independent evaluators at baseline (before treatment was initiated) and at the 9-month follow-up. These widely used clinical scales continue to be applied in contemporary research, as demonstrated by recent publications [89,90,91]. For the PANSS, the three traditional sub-scales were considered: positive symptoms, negative symptoms and general psychopathology. A modified version of the Nottingham Onset Schedule (NOS) [92] was administered to assess the duration of untreated psychosis (DUP).

Since FEP is generally a phase of high diagnostic instability, the specific ICD-10 codes for psychosis (F1x.4; F1x.5; F1x.7; F20–29; F30.2, F31.2, F31.5, F31.6, F32.3, F33.3) were assigned after 9 months. The best-estimate ICD-10 diagnoses were made by consensus of a panel of clinicians by considering all the available information gathered since the enrolment into the study, as required to apply the Item Group Checklist (IGC) of the Schedule for Clinical Assessment in Neuropsychiatry [93,94]. Forty-six (24.6%) patients received a diagnosis of schizophrenia (SCZ, ICD-10 F20) and 47 (24.7%) of affective psychosis (AP, ICD-10 F30, F31, F32), while 97 (50.8%) received other diagnoses (non-affective psychosis, ICD-10 F21, F22, F23, F25, F29, F10, F12, F19).

At baseline, 13 patients were drug-free, whereas 161 (84.3%) were taking antipsychotics, 23 (12.0%) mood stabilisers, 35 (18.3%) antidepressants, and 47 (24.6%) anxiolytics. The total number exceeds the number of patients because some of them may have been taking more than one type of medication.

### 4.2. Treatment

The experimental treatment (EXP) consisted of a multi-element psychosocial intervention, in addition to routine care. This included cognitive–behavioural therapy for psychosis for patients [95,96] and psychosis-focused family intervention [97] for families, accompanied by case management [98] involving both patients and their families. Treatment was provided by the CMHC staff, trained in the previous 6 months and supervised by experts. The intervention began when patients achieved clinical stabilisation (i.e., a condition in which they could collaborate in a brief clinical examination). Core baseline measures were collected. CMHCs in the control arm provided only treatment as usual (TAU) which, in Italy, includes personalised outpatient psychopharmacological treatment and non-specific supportive clinical management by the CMHC [99]. In the TAU group, family interventions consisted of non-specific informal support sessions. Treatment has been described in detail in the study reporting the results of the GET UP PIANO clinical trial [50]. The EXP group included 128 patients, whereas the TAU group included 63. 

At the end of treatment, considering the whole group (*n* = 191), 38 patients (19.9%) were not taking any psychotropic medication, whereas 148 (77.5%) were taking antipsychotics, 28 (14.7%) mood stabilisers, 57 (29.8%) antidepressants, and 78 (40.8%) anxiolytics.

### 4.3. Analysis of Biological Markers

Peripheral venous blood was drawn from each subject in anticoagulant-free tubes in the morning, after an overnight fast (between 07:00 and 10:00 a.m.). The tubes were kept at room temperature for 2 h followed by 1 h at 4 °C, before serum separation by centrifugation (1620× *g* for 15 min). Serum samples were then stored at −80 °C until the time of assays. All serum samples were collected and processed according to standardised procedures across CMHCs and subsequently analysed centrally using the same assays.

All the details about the procedures and assays used to determine the serum levels of the 19 candidate analytes are provided in Supplementary File S1; all the samples and standards were measured in duplicate.

### 4.4. Statistical Analysis

Data are described as mean ± standard deviation (SD) for continuous variables or as frequencies and percentages for categorical ones. Gaussianity assumption for continuous variables was assessed by the Kolmogorov–Smirnov and Shapiro–Wilk tests, combined with visual inspection.

Concentration values of the biomarkers that were below the assay limit of detection (LOD) were excluded from the analyses; this approach was chosen to avoid potential bias associated with the arbitrary imputation of non-quantifiable values.

Comparisons between groups were carried out through *t*-tests for normally distributed variables and Mann–Whitney tests for non-normally distributed ones, whereas correlations were evaluated through Pearson’s r coefficient or Spearman’s ρ coefficient, depending on data normality.

Clinical scale changes across treatment were assessed through analysis of covariance (ANCOVA) with repeated measures.

The predictive effect of baseline biomarker levels on changes in clinical scale scores was examined using ANCOVA models, where the scores at T1 were the dependent variables, while baseline scores, baseline biomarker levels, treatment group (EXP vs. TAU) and their interaction (biomarker × treatment group) were entered as fixed effects. The relationships between changes in biomarker levels and changes in clinical scores were similarly assessed, treating biomarker levels and clinical scores at T1 alternately as dependent variables. Each model included baseline level of the variable, dichotomous change in the other variable (increased or decreased), treatment group and their interaction (change in the other variable × treatment group) as fixed effects.

Gender and age of onset were included as covariates in all the analyses to control for potential confounding.

Generalised linear mixed models (GLMMs) were used to evaluate the moderating effect of treatment on longitudinal changes in biomarker levels, including only patients with available measurements at both T0 and T1. Each biomarker was analysed as a repeated dependent variable over time, with time, treatment group and their interaction included as fixed effects, and gender and age of onset as covariates. A subject-level random intercept was included to account for repeated measurements. The distribution family and link function for each biomarker were selected based on its underlying distribution, and residual diagnostics were conducted to assess model fit. Estimated marginal means, along with standard errors and effect sizes, were then reported. As a sensitivity analysis, a random intercept for CMHC was included to assess the extent of variability explained by the recruiting centre.

Since this study was designed as an exploratory investigation of possible inflammatory and metabolic changes associated with the first phase of treatment in FEP patients, no single biomarker was predefined as a primary endpoint; all 19 biomarkers were treated as equally important and evaluated as exploratory outcomes. Thus, no formal power calculation was performed.

Statistical analyses were carried out by SPSS 26.0 and through R language v.4.3.2 with the package “glmmTMB” for generalised linear mixed models. Statistical significance was set at *p* < 0.05.

## 5. Conclusions

In conclusion, findings of this exploratory study partially support the research hypothesis. Treatment was associated with changes in a subset of metabolic biomarkers, which are also implicated in critical brain processes, but no significant effects were detected for inflammatory biomarkers. Additionally, no baseline biomarker showed a predictive effect on clinical outcomes, and no significant correlations were found between changes in biomarker levels and clinical improvements across treatment. These results indicate that while the interventions could influence certain metabolic pathways, the investigated biomarkers do not appear to have utility in predicting treatment response.

To disentangle the relative contribution of disease progression versus possible treatment effects, future studies should include patients with first-episode psychosis who are completely drug-free, and also evaluate the effects of specific antipsychotic medications known to have more pronounced metabolic effects, that is, atypical antipsychotics, as well as individual drugs such as clozapine. These results pave the way for future studies aimed at unravelling the mechanisms underlying the explored interventions and at identifying associated biomarkers, ultimately advancing the precision and personalisation of therapeutic strategies for psychoses.

## Figures and Tables

**Table 1:** Socio-demographic and anthropometric characteristics of the whole study cohort and of the EXP and TAU groups with corresponding statistical tests.

	Whole Cohort (*n* = 191)	EXP Group (*n* = 128)	TAU Group (*n* = 63)	*p*-Value EXP vs. TAU
Sex (% males, % females)	54.5% M; 45.5% F	60.2% M; 39.8% F	42.9% M; 57.1% F	**0.024**
Age of onset (mean ± SD; years)	30.0 ± 9.9	28.9 ± 9.9	32.1 ± 9.6	**0.025**
BMI (mean ± SD)	23.6 ± 3.3	23.6 ± 3.4	23.7 ± 3.1	0.874

**Table 2:** Baseline concentrations of the 19 biomarkers in the whole study cohort (means ± standard deviations).

Biomarker	Concentration (Mean ± SD)
BDNF ng/mL	41.7 ± 9.33
Leptin ng/mL	10.1 ± 8.55
RANTES/CCL5 ng/mL	27.6 ± 14.6
IL-1RA ng/mL	1.11 ± 0.71
VEGF pg/mL	195.6 ± 94.9
MIP-1b/CCL4 pg/mL	153.1 ± 77.6
IL-6 pg/mL	4.55 ± 7.42
IL-8 pg/mL	19.6 ± 32.3
IL-10 pg/mL	7.41 ± 18.6
IL-17 pg/mL	9.89 ± 29.1
C-peptide pg/mL	706 ± 376
Ghrelin ng/mL	1.29 ± 0.47
GIP pg/mL	203 ± 457
GLP-1 pg/mL	377 ± 83.3
Glucagon pg/mL	908 ± 288
Insulin pg/mL	360 ± 236
PAI-1 ng/mL	42.9 ± 23.1
Resistin ng/mL	3.66 ± 2.07
Visfatin ng/mL	4.52 ± 5.31

Abbreviations: BDNF: Brain-Derived Neurotrophic Factor; RANTES/CCL5: C-C motif Chemokine Ligand 5; IL-1RA: Interleukin 1 Receptor Antagonist; VEGF: Vascular Endothelial Growth Factor; MIP-1b/CCL4: C-C motif Chemokine Ligand 4; IL-6: Interleukin 6; IL-8: Interleukin 8; IL-10: Interleukin 10; IL-17: Interleukin 17; GIP: Gastric Inhibitory Polypeptide; GLP-1: Glucagon-Like Peptide-1; PAI-1: Plasminogen Activator Inhibitor-1.

**Table 3:** Results of the analysis of covariance (ANCOVA) with repeated measures and means ± standard deviations for the three clinical scale scores in the EXP and TAU groups at baseline (T0) and after 9 months of treatment (T1).

	*p*-Value Time Effect	*p*-Value Time × Treatment Group Effect	Mean ± SD EXP Group (*n* = 128)		Mean ± SD TAU Group (*n* = 63)	
			T0	T1	T0	T1
Mean Tot. PANSS	**<0.001**	**0.008**	2.37 ± 0.59	1.62 ± 0.54	2.17 ± 0.53	1.73 ± 0.61
HAM-D	**<0.001**	**0.010**	17.22 ± 6.87	8.48 ± 6.60	14.70 ± 6.56	9.51 ± 6.91
GAF	**<0.001**	**0.035**	43.34 ± 12.15	63.67 ± 16.38	46.06 ± 13.05	59.63 ± 17.16

**Table 4:** Levels of leptin, ghrelin and GLP-1 (estimated marginal means ± standard errors) at baseline (T0) and at the end of treatment (T1) in the EXP and TAU groups, with relative sample sizes (N), effect sizes (ES) with 95% confidence intervals, and *p*-values for time effects (*p* time) and time × treatment group effects (*p* time × treatment group). ESs represent model-based percent changes derived from exponentiated contrasts of the Gamma-log GLMM.

	EXP Group				TAU Group				
	*n*	Mean ± SE T0	Mean ± SE T1	*p* Time	*n*	Mean ± SE T0	Mean ± SE T1	*p* Time	*p* Time *×* Treatment Group
Leptin (ng/mL)	56	7.68 ± 0.79	10.59 ± 1.10	0.003	25	9.76 ± 1.50	12.93 ± 2.00	0.084	0.839
		ES: +37.9% [+11.4%, +71%]				ES: +32.5% [−3.7%, +81%]			
Ghrelin (ng/mL)	58	1.21 ± 0.05	1.06 ± 0.05	0.001	25	1.18 ± 0.08	1.18 ± 0.08	0.966	0.074
		ES: −12.4% [−5%, −19.4%]				ES: 0% [−21.5%, +14%]			
GLP-1 (pg/mL)	58	331.76 ± 8.36	312.88 ± 7.88	<0.001	24	355.26 ± 13.96	332.46 ± 13.07	0.012	0.805
		ES: −5.7% [−2.5%, −8.8%]				ES: −6.4% [−1.5%, −11.1%]			

## Data Availability

The data obtained in the study cannot be made publicly available for privacy and ethical reasons.

## Supplementary Materials

The following supporting information can be downloaded at: https://www.mdpi.com/article/10.3390/ijms27042065/s1.

## Abbreviations

The following abbreviations are used in this manuscript:

FEP	First-episode psychosis
EXP	Experimental treatment
TAU	Treatment as usual
GLP-1	Glucagon-like peptide 1
