# Supplementary material for: Exploration of Early-Treatment-Associated Changes in Metabolic and Inflammatory Biomarkers in First-Episode Psychosis in Italian Patients
Source: Int J Mol Sci. 2026 Feb 23;27(4):2065. doi: 10.3390/ijms27042065 (PMC12940655; doi:10.3390/ijms27042065)
Supplement: Supplementary file 1 [file ijms-27-02065-s001.zip › Supplementary File S1.pdf]

C-peptide, **ghrelin**, **glucagon**, **GIP**, **GLP-1**, **insulin**, **leptin**, **PAI-1**, **resistin**, **visfatin** were analysed by using the Bio-Plex Pro™ Human Diabetes kit (Bio-Rad, CA, USA).
IL-6, **IL-8**, **IL-10**, **IL-17** were analyzed by using the Bio-Plex Pro™ Human Cytokine custom kit (Bio-Rad, CA, USA).
For both panels, 12.5 µl of sample were diluted 1:4 with the sample diluents, and 50 µl of the diluted sample were incubated in a pre-wet filter plate for 1 hour in the dark with the biotinylated detection antibody. Each analyte was detected by the addition of a streptavidin-phycoerythrin solution and quantified using the Bio-Plex 200 System array reader (Bio-Rad, CA, USA). Standard curves were created using the reference standard samples supplied by the manufacturer. Analyte concentrations were calculated using the Bio-Plex Manager software 6.0.

BDNF was measured through an ELISA method using the Human BDNF Kit (R&D System, MN, USA), according to the manufacturer's instructions. Serum samples were diluted 1:100.

RANTES/CCL5, **VEGF**, **IL-1RA**, **MIP-1b/CCL4** were measured by Luminex Multianalyte Service (LaboSpace, Milan, Italy).
