# Supplementary material for: Exploration of Early-Treatment-Associated Changes in Metabolic and Inflammatory Biomarkers in First-Episode Psychosis in Italian Patients
Source: Int J Mol Sci. 2026 Feb 23;27(4):2065. doi: 10.3390/ijms27042065 (PMC12940655; doi:10.3390/ijms27042065)
Supplement: Supplementary file 1 [file ijms-27-02065-s001.zip › Supplementary File S2.pdf]

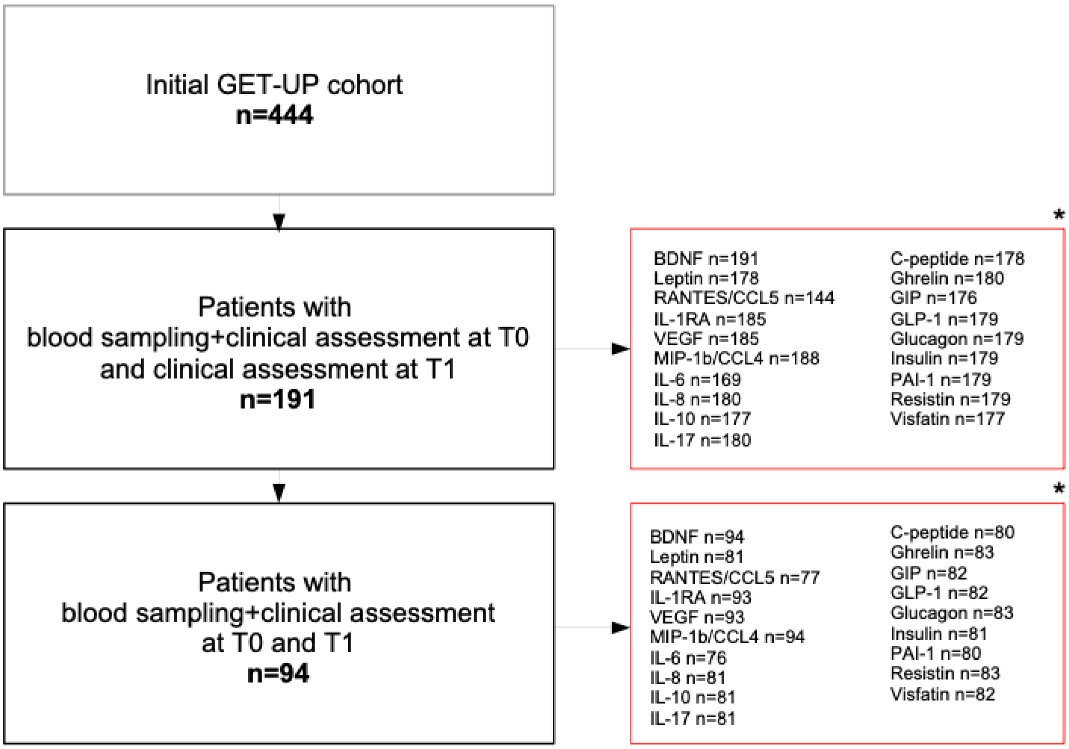

* The number of patients varies across analytes due to concentrations below the limit of detection (LOD).
